# Heart Failure Treatment in Underserved Populations: A Comprehensive Analysis of Sacubitril/Valsartan and Sodium-Glucose Transporter 2 Inhibitor (SGLT2i) Prescription Trends at a Safety Net Hospital

**DOI:** 10.7759/cureus.81757

**Published:** 2025-04-05

**Authors:** Khalid Sawalha, Andrew J Fancher, Subhi Al'Aref, Angel Lopez Candales

**Affiliations:** 1 Cardiovascular Disease, University of Arkansas for Medical Sciences, Little Rock, USA; 2 Cardiometabolic Medicine, University of Missouri-Kansas City School of Medicine, Kansas City, USA; 3 Internal Medicine, University of Kansas School of Medicine-Wichita, Wichita, USA; 4 Cardiology, University of Arkansas for Medical Sciences, Little Rock, USA; 5 Cardiovascular Medicine, University of Puerto Rico School of Medicine, San Juan, PRI

**Keywords:** heart failure, sacubitril/valsartan, safety-net hospital, sglt2i use, underserved population

## Abstract

Background

Sacubitril/valsartan and sodium-glucose transporter 2 inhibitors (SGLT2is) are emerging classes of medications that have become key components of guideline-directed medical therapy for patients with heart failure (HF) with reduced ejection fraction and New York Heart Association class II, III, or IV symptoms. Both sacubitril/valsartan and SGLT2is have demonstrated the ability to improve morbidity and mortality in HF patients. This analysis evaluates current prescribing trends of sacubitril/valsartan and SGLT2is in a safety net hospital setting.

Methods

In this retrospective study, a chart review was conducted to identify sacubitril/valsartan use, categorized by drug dose and prescriber specialty, at University Health Truman Medical Centers in Kansas City, Missouri, USA, from October 1, 2021, to October 31, 2022. A second chart review similarly identified SGLT2i usage, also categorized by drug dose and prescriber specialty, within the same time frame and healthcare system.

Results

Of the 769 patients prescribed sacubitril/valsartan, 497 (64.6%) were prescribed the 24 mg/26 mg dose, 193 patients (25.1%) received the 49 mg/51 mg dose, and 79 patients (10.3%) were on the 97 mg/103 mg dose. Cardiologists accounted for only 23.3% of sacubitril/valsartan prescriptions, while ancillary staff, including nurse practitioners, physician assistants, and pharmacists, accounted for the majority (49.8%) of prescriptions. Regarding SGLT2is, 2,287 patients were prescribed these medications: 343 patients were prescribed dapagliflozin (188 at the 5 mg dose and 155 at the 10 mg dose); one patient received ertugliflozin at the 5 mg dose; 634 patients were prescribed canagliflozin (404 at the 100 mg dose, 173 at the 300 mg dose, 40 at the 50 mg dose with metformin combination, and 17 at the 150 mg dose with metformin combination); and 1,309 patients were prescribed empagliflozin (972 at the 10 mg dose, 333 at the 25 mg dose, two at the 5 mg dose with metformin combination, and two at the 12.5 mg dose with metformin combination). Cardiologists prescribed only 10.0% of SGLT2is, while internal/family medicine physicians accounted for the majority (52.3%) of prescriptions.

Conclusions

Robust evidence supports the use of sacubitril/valsartan and SGLT2is in HF, with both also proving effective for treating other diseases commonly coexisting with HF. The observed dosing patterns and distribution of prescribers reflect the broad utility of these novel therapeutics in diverse clinical settings.

## Introduction

Heart failure (HF) continues to be a significant global health challenge, necessitating innovative therapeutic approaches [1]. Sacubitril/valsartan, a medication introduced in 2015 for the treatment of HF with reduced ejection fraction (HFrEF), has proven effective in reducing morbidity and mortality associated with HF. The Prospective Comparison of ARNI with ACEI to Determine Impact on Global Mortality and Morbidity in Heart Failure (PARADIGM-HF ) trial highlighted its efficacy in improving outcomes, marking a major advancement in HF management [2]. The recommended initial dose is 49/51 mg twice daily, with gradual escalation to the intended maintenance dose of 97/103 mg twice daily [2].

Similarly, sodium-glucose transporter 2 inhibitors (SGLT2is), a newer class of medications introduced in 2013, have shown significant benefits in improving HF morbidity and mortality. Several randomized controlled trials involving patients with type 2 diabetes mellitus (DMII) and established cardiovascular disease have demonstrated that these drugs can help prevent HF hospitalizations [3-5]. This led to the initiation of the Dapagliflozin and Prevention of Adverse Outcomes in Heart Failure (DAPA-HF) and EMPagliflozin outcomE tRial in Patients With Chronic Heart Failure With Reduced Ejection Fraction (EMPEROR-Reduced) trials. These studies revealed substantial improvements in hospitalizations and mortality rates for patients with HFrEF [6-8], further advancing treatment options for HF. Currently, the recommended dose of dapagliflozin for HF is 10 mg daily, with the initial dose for DMII set at 5 mg, which can be increased to 10 mg if tolerated [6,9,10]. Empagliflozin, for HF, is also recommended at 10 mg daily, with the initial dose for DMII set at 10 mg, increasing to 25 mg daily if necessary [11,12].

In 2022, the American College of Cardiology/American Heart Association/Heart Failure Society of America (ACC/AHA/HFSA) guidelines incorporated sacubitril/valsartan into the treatment options for HF with moderate reduction in ejection fraction (LVEF 41-49%) and for specific patients with HF and preserved ejection fraction (LVEF ≥50%) [11]. This recommendation is especially relevant for individuals with a left ventricular ejection fraction near the lower range of the HF with preserved ejection fraction spectrum. These guidelines were introduced following the PARAGON-HF trial, which demonstrated that individuals with HF and preserved ejection fraction, particularly those with ejection fractions below 57% and those of female gender, could potentially benefit from sacubitril/valsartan treatment, especially when evidence-based therapies were otherwise inaccessible [13]. However, in real-world settings, only one in five sacubitril/valsartan prescriptions dispensed in 2018-2019 corresponded to the target dose of 97/103 mg, with even lower utilization observed among the elderly population [14]. While the number of prescriptions issued by non-cardiologists has increased significantly, higher doses were more frequently prescribed by cardiologists [14].

Similarly, in 2022, SGLT2is were included in the guidelines for the treatment of symptomatic chronic HFrEF as a 1A recommendation to reduce HF hospitalizations and cardiovascular mortality, regardless of the presence of DMII [11]. This guideline update was informed by the results of the DAPA-HF and EMPEROR-Reduced trials, which showed that SGLT2is reduced the composite of cardiovascular death or HF hospitalization by 25% compared to placebo [6-8]. SGLT2i prescriptions have risen steadily since their introduction, with one study reporting 63.2 million prescriptions between January 2015 and December 2020; however, cardiologists represented only 1.5% of these prescriptions [15].

As these medications have recently been incorporated into HF guideline-directed medical therapy (GDMT), it is crucial to analyze prescribing patterns for sacubitril/valsartan and SGLT2is across various medical specialties. Additionally, these novel medications remain significantly more expensive than other GDMT options. Our study aimed to analyze current trends in the prescription of these newer treatments within a large, multidisciplinary healthcare system. Specifically, our investigation focused on a safety net hospital serving the Kansas City metro area, which primarily treats an underserved population. This context allowed us to explore how these costly medications are being prescribed within a healthcare system where a substantial portion of the population faces socioeconomic challenges.

## Materials and methods

Study design

To investigate utilization trends in the prescription of novel HF GDMT in a safety net hospital, a retrospective analysis was conducted using prescription data from the University Health System (UHS) in Kansas City, Missouri. A report detailing all prescriptions for sacubitril/valsartan and SGLT2is within a specified time frame was generated. Data collected included prescriber name, specialty, drug dose, and formulation. These medications were chosen as they represent newer pillars of HF GDMT. The study aimed to explore who was prescribing these therapies and the patterns in their prescribing behaviors. Specifically, the study focused on how these novel and expensive therapeutics were being used in an underserved population.

Study population and sample size

The study population consisted of all patients who may have been prescribed sacubitril/valsartan or SGLT2is within UHS, encompassing both inpatient and outpatient settings across a broad, heterogeneous healthcare system. UHS serves as a critical safety net hospital in the Kansas City metro area, predominantly providing care to an underserved population. Approximately 66% of the patient population is enrolled in Medicare or Medicaid or is uninsured. UHS provides nearly $150,000,000 in uncompensated care annually, making this study population an excellent representation of the larger underserved demographic in the US. We selected prescription data from October 1, 2021, to October 31, 2022, which included prescriptions for 769 patients during that time. Given that these novel therapies were recently integrated into GDMT, we focused on prescriptions from this year, as those prior to this period were unlikely to have been intended for HF treatment.

Study measures and statistical analysis

A retrospective analysis was conducted to identify instances of sacubitril/valsartan and SGLT2i utilization across various specialties and providers. Data from 769 patients prescribed sacubitril/valsartan and 2,287 patients prescribed SGLT2is between October 1, 2021, and October 31, 2022, was collected. The data included prescriber name and specialty, patient medical record number (MRN), medication prescribed, and dose. MRNs were used to eliminate duplicate prescriptions, and these identifiers were then removed from the dataset before further analysis. Using Microsoft Excel (Microsoft Corporation, Redmond, WA, USA), the number of prescriptions for each medication and dose was counted across different specialties. The specialties observed included weight management, nephrology, endocrinology, internal medicine, family medicine, cardiology, nurse practitioners (NPs), physician assistants (PAs), and pharmacists. All other specialties were grouped into a “miscellaneous/other” category. NPs, PAs, and pharmacists were categorized together as “ancillary.” Internal medicine and family medicine were combined into a “primary and general care” category. The number of prescriptions for each dose was divided by the total number of prescriptions for each medication to calculate dose percentages. The number of prescriptions from each specialty was divided by the total number of prescriptions for that medication to determine the percentage of total prescribers from each specialty.

Ethics statement

This study performed a retrospective analysis of data previously collected by the outpatient cardiology clinics. It was exempt from a full institutional review board (IRB) review, as it involved secondary analysis of anonymized data. The study was conducted in accordance with the Declaration of Helsinki principles for medical research involving archived records. Patient identifiers, other than the unique MRN, were not collected in the initial report and were destroyed as soon as possible, as mentioned above. Although the study was exempt from full IRB oversight, it adhered to guidelines set out by the University of Missouri-Kansas City IRB to ensure patient confidentiality. Best practices for protecting patient confidentiality in data storage and access were observed. The research team has no conflicts of interest to disclose.

## Results

**Figure 1:**
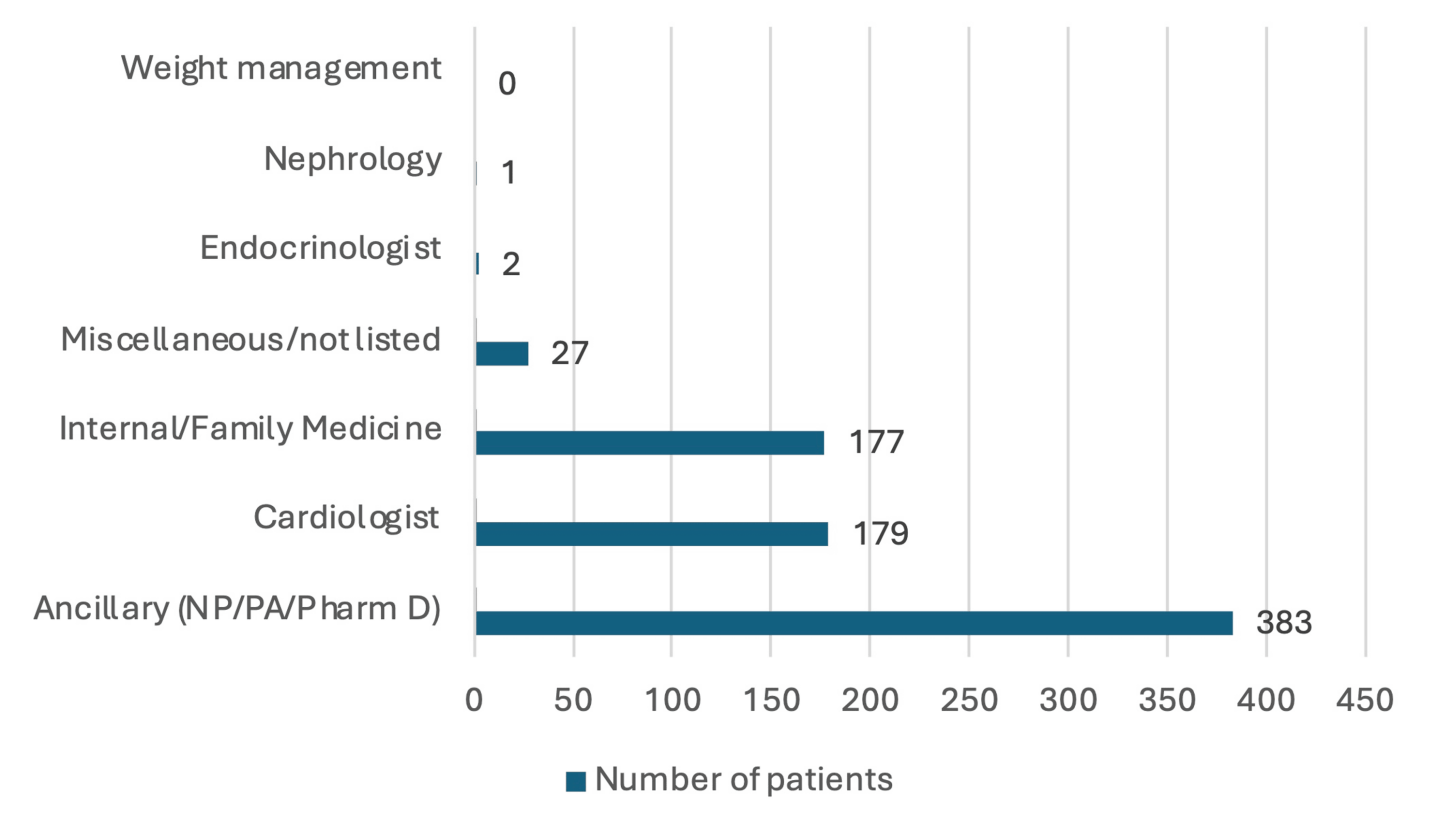
Sacubitril/valsartan prescribers by specialty NP, nurse practitioner; PA, physician assistant

The distribution of prescriptions for sacubitril/valsartan by prescriber specialty was as follows: one patient (0.1%) was prescribed sacubitril/valsartan by a nephrologist, two patients (0.3%) were prescribed the medication by endocrinologists, and 27 patients (3.5%) were prescribed it by miscellaneous or unlisted specialties. Internal and family medicine physicians prescribed sacubitril/valsartan to 177 patients (23.0%), while 179 patients (23.3%) received prescriptions from cardiologists. The majority of prescriptions, 383 patients (49.8%), were issued by NPs, PAs, or pharmacists (Figure 1).

**Figure 2:**
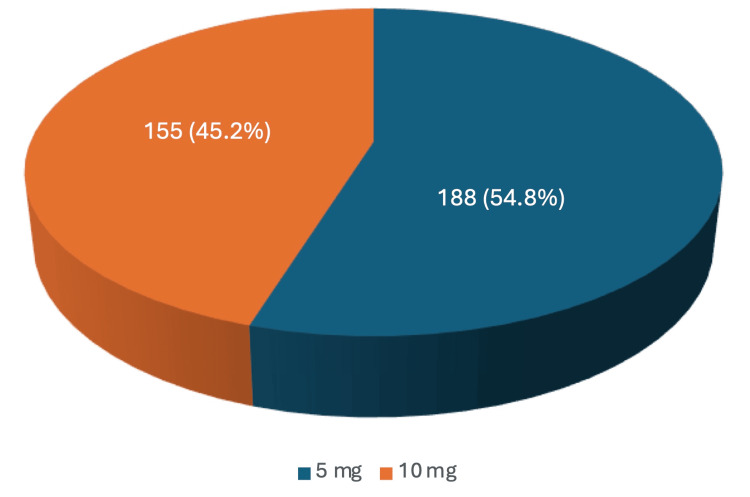
Dapagliflozin doses and percentage of utilization

**Figure 3:**
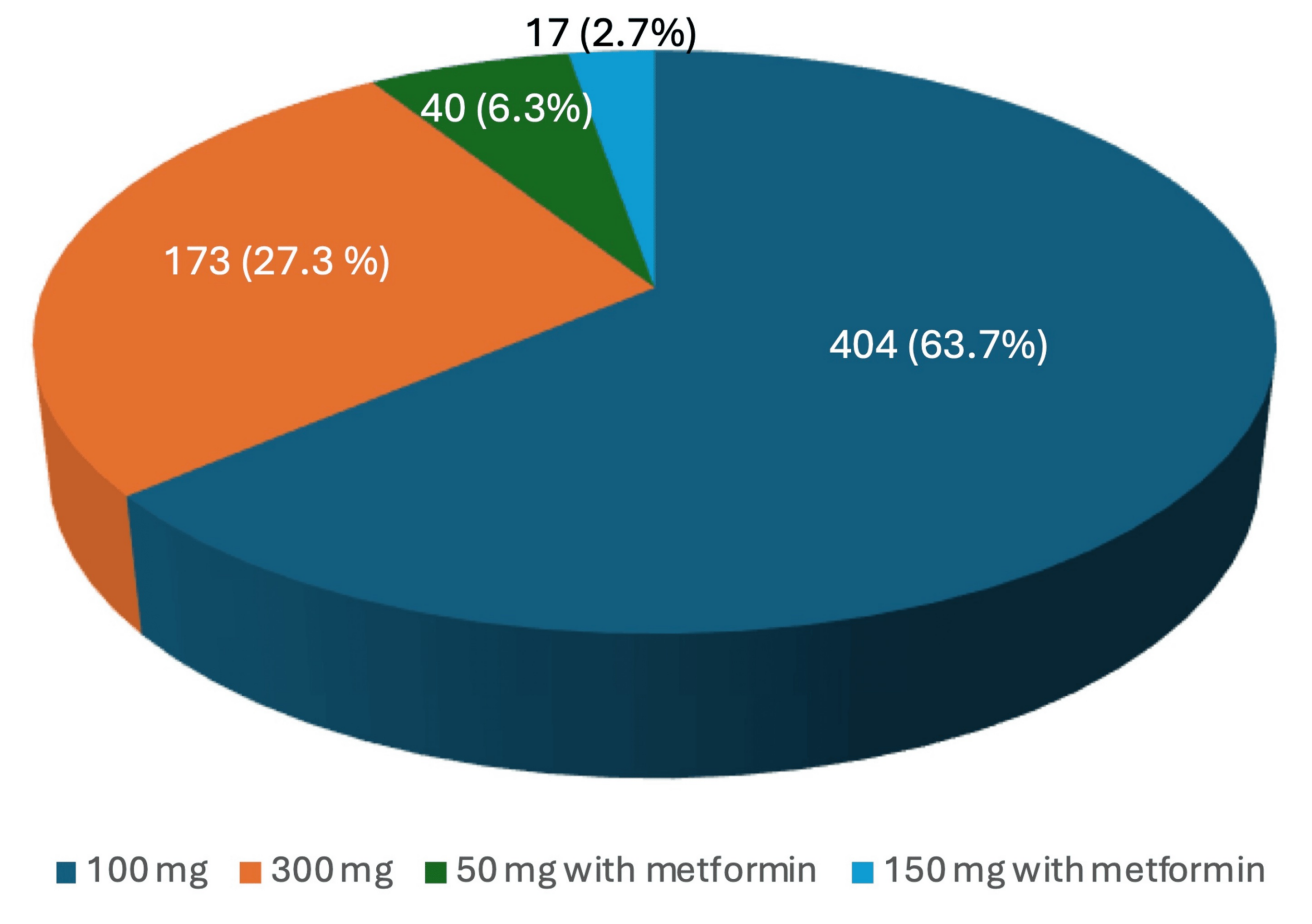
Canagliflozin doses and percentage of utilization

**Figure 4:**
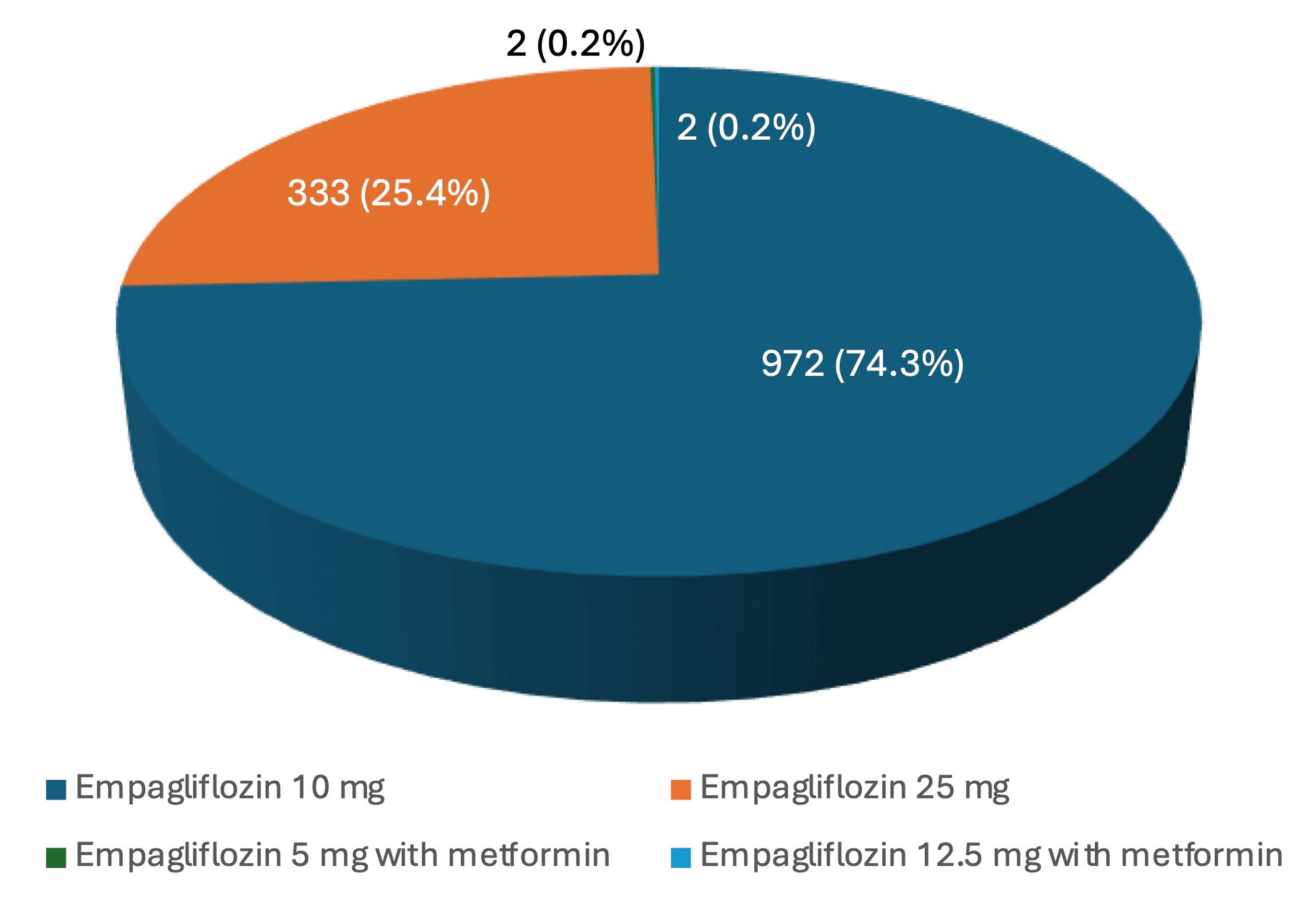
Empagliflozin doses and percentage of utilization

The retrospective review of SGLT2i utilization identified 4,465 instances of SGLT2i prescriptions, with 2,178 representing duplicate prescriptions for refills. After excluding duplicates, 2,287 patients were prescribed SGLT2is within the UHS during the specified time frame. Among them, 343 patients were prescribed dapagliflozin, with 188 (54.8%) receiving the 5 mg dose and 155 (45.2%) receiving the 10 mg dose. One patient was prescribed ertugliflozin at a 5 mg dose. A total of 634 patients were prescribed canagliflozin, with 404 (63.7%) receiving the 100 mg dose, 173 (27.3%) the 300 mg dose, 40 (6.3%) a 50 mg dose in combination with metformin, and 17 (2.7%) a 150 mg dose in combination with metformin. Additionally, 1,309 patients were prescribed empagliflozin, with 972 (74.3%) receiving the 10 mg dose, 333 (25.4%) the 25 mg dose, 2 (0.2%) the 5 mg dose in combination with metformin, and 2 (0.2%) the 12.5 mg dose in combination with metformin. The percentage utilization of each dose for each unique drug is visually represented in Figure 2, Figure 3, and Figure 4 (excluding ertugliflozin, which had only one prescription).

**Figure 5:**
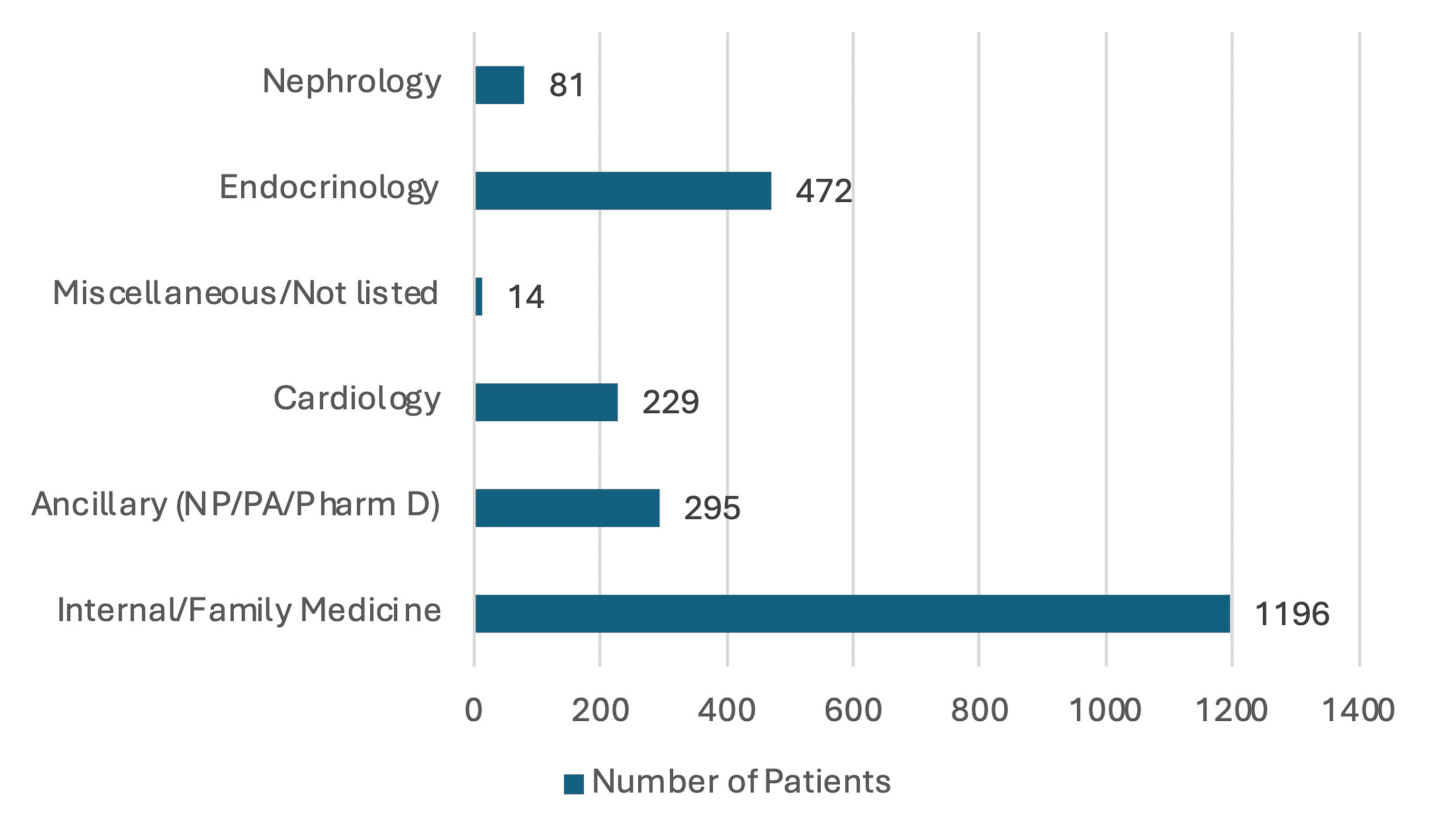
SGLT2i prescriptions by specialty NP, nurse practitioner; PA, physician assistant; SGLT2i, sodium-glucose transporter 2 inhibitor

The distribution of SGLT2i prescriptions by prescriber specialty was as follows: 81 (3.5%) patients received SGLT2is from nephrologists; 472 (20.6%) from endocrinologists; 14 (0.6%) from miscellaneous or unlisted specialties; 229 (10%) from cardiologists; 295 (12.9%) from NPs, PAs, or PharmDs; and 1,196 (52.3%) from internal/family medicine physicians. These results are visually represented in Figure 5.

## Discussion

The introduction of sacubitril/valsartan and SGLT2is as therapeutic options for HF has sparked considerable interest and raised critical considerations within cardiovascular medicine [16]. Our retrospective analysis provides insight into several key aspects of how these novel HF therapies are utilized among a diverse cohort of patients, contributing valuable information to the ongoing discussion on their clinical application.

The dosing patterns observed in our study highlight a broad spectrum of treatment strategies employed by healthcare providers. Notably, the majority of patients receiving sacubitril/valsartan (65%) were prescribed the 24 mg/26 mg dose, consistent with the recommended starting dose for those who have not previously used angiotensin-converting enzyme inhibitors or angiotensin receptor blockers. A substantial portion of patients (25%) received the 49 mg/51 mg dose, likely indicating those who tolerated the initial dose well and transitioned to a higher dose as guided by their clinical response. Interestingly, a smaller subset (10%) was prescribed the 97 mg/103 mg dose, possibly reflecting specialized cases or a cautious approach by providers in specific clinical scenarios.

For SGLT2is, our study found that 45.2% of patients receiving dapagliflozin were prescribed the 10 mg daily dose indicated for HFrEF. However, the fact that dapagliflozin is also indicated for DMII with an initial dose of 5 mg daily, which can be increased to 10 mg as tolerated, complicates this finding. Similarly, 74.3% of patients receiving empagliflozin were prescribed the 10 mg daily dose for HFrEF, although this drug is also indicated for DMII with an initial 10 mg daily dose, which can be increased to 25 mg as tolerated. Additionally, ertugliflozin and canagliflozin are not currently FDA-approved treatments for HFrEF and lack dedicated dosages for this condition. Nonetheless, growing evidence, such as the Canagliflozin and Renal Events in Diabetes with Established Nephropathy Clinical Evaluation (CREDENCE) trial for canagliflozin, suggests that these drugs also reduce the risk of hospitalization and cardiovascular death in HF patients, given their similar mechanisms of action [17].

A limitation of our study is that the retrospective design collected data on instances of SGLT2i prescriptions, including drug, dosage, and prescriber, but did not specify the intent behind the prescriptions. As a result, we were unable to determine how many patients were prescribed SGLT2is for HFrEF versus DMII. The overlap between these patient populations further complicates this distinction. For instance, a patient with both DMII and HFrEF may receive the DMII dose of dapagliflozin while still benefiting from the drug’s effects on HFrEF. Given that recent guidelines have incorporated data on SGLT2is, further research is needed to explore the benefits of different SGLT2is and dosages for HFrEF, as higher doses, similar to trends seen in DMII treatment, may be more beneficial for these patients.

The distribution of prescriber specialties offers interesting insights into the evolving landscape of HF management and the role of novel therapeutic agents. While cardiology has traditionally been central to HF treatment, our findings show that only 23.3% of sacubitril/valsartan prescriptions and 10% of SGLT2i prescriptions originated from cardiologists. This could indicate a shift toward greater multidisciplinary collaboration, with ancillary staff providers and primary care practitioners playing a more substantial role in managing HF patients. The prominence of NPs, PAs, and pharmacists as sacubitril/valsartan prescribers (49.8%) and internal/family medicine practitioners as SGLT2i prescribers (52.3%) highlights the growing involvement of multidisciplinary teams in optimizing HF therapy. Part of this shift may be due to increased accessibility and the expanded role of primary care providers and advanced practice providers in patient care, especially beyond traditional outpatient visits. Moreover, this pattern may reflect the broad utility of these novel therapeutics, which are often used in the treatment of conditions like DMII and chronic kidney disease, both of which frequently coexist with HF. Given this overlap, it is logical that many prescriptions come from primary care providers, who are likely using these medications for their synergistic effects in treating multiple diseases.

Another potential explanation for the prescriber distribution we observed may be linked to efforts at University Health Truman Medical Centers to make these costly, novel HF therapies more accessible to patients. As a safety net hospital system serving a largely underserved population, many of whom lack prior insurance or healthcare access, University Health provides a medication assistance program that offers free prescriptions to patients meeting specific criteria. This system also offers 340B pricing for outpatient prescriptions for eligible patients, helping to make novel therapeutics more affordable. However, this may be influencing the distribution of prescriptions, as the logistical work of facilitating prescriptions might fall to ancillary staff or primary care providers due to the time constraints faced by cardiologists.

Our study underscores the need for continued investigation into the factors influencing prescriber choices and their impact on patient outcomes. Further research is necessary to better understand the rationale behind the observed dosing strategies and prescriber distributions. Additionally, assessing the long-term clinical benefits and potential adverse effects of sacubitril/valsartan and SGLT2is across different patient profiles remains an essential area for future exploration.

As noted, our study has several limitations, including its retrospective nature and single-center design, which limits the generalizability of the findings to other settings. A collaborative multicenter study involving a larger, more diverse patient population is needed to better understand demographic and geographic variations in the use of novel GDMTs in HF management.

## Conclusions

Our retrospective analysis provides valuable insights into the utilization of sacubitril/valsartan and SGLT2is in HF treatment. Two key conclusions emerge from our findings. First, primary care providers represent the majority of prescribers for sacubitril/valsartan and SGLT2is, possibly due to the timing of our data collection, which occurred before these medications were officially adopted into GDMT for HF by the ACC/AHA/HFSA. This trend may also reflect the wide utility of these drugs in synergistically managing other conditions frequently coexisting with HF. Second, the observed dosing patterns and prescriber distribution highlight the evolving landscape of HF management, characterized by the integration of novel therapeutic approaches and the increasing involvement of multidisciplinary healthcare teams. As we continue to navigate the dynamic field of cardiovascular medicine, further research into these novel medications is crucial to guide evidence-based decision-making and optimize patient outcomes in HF treatment.
